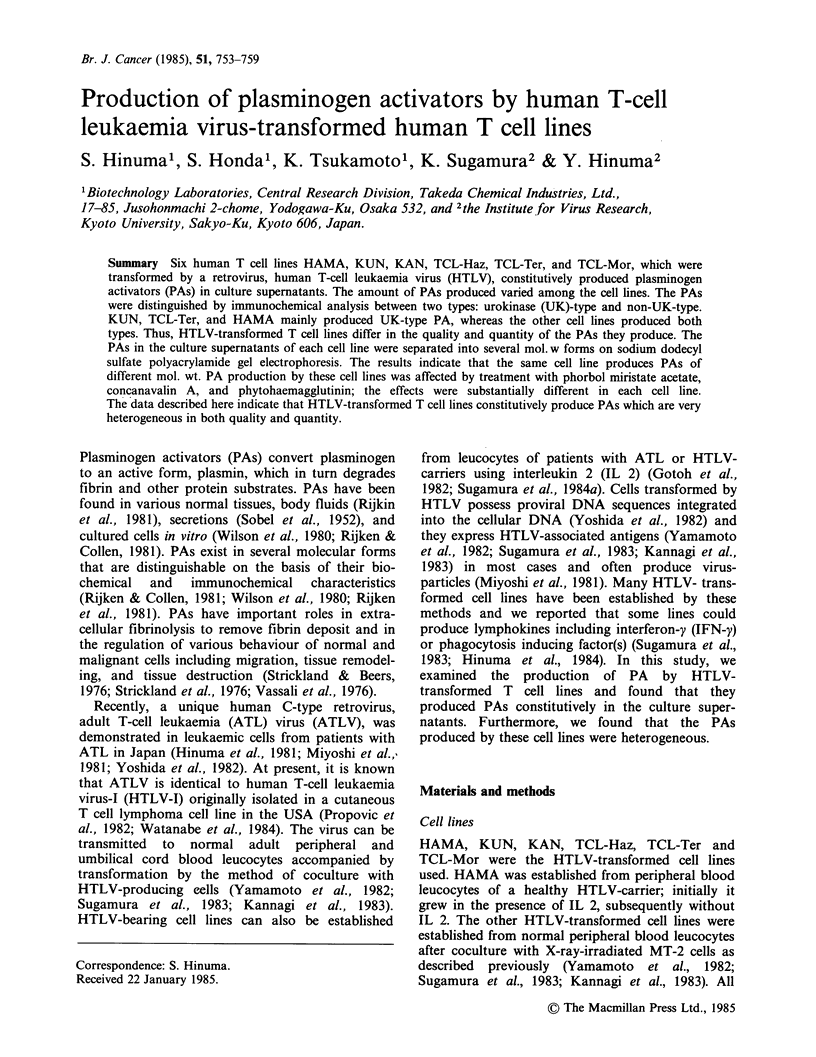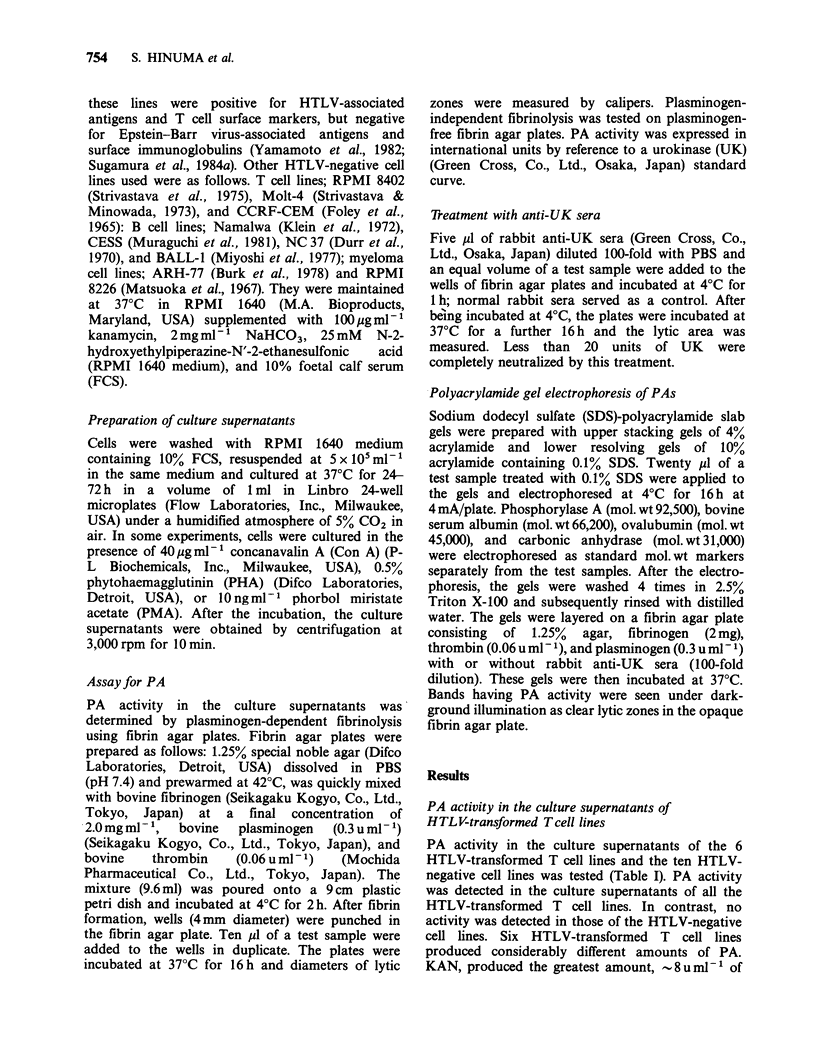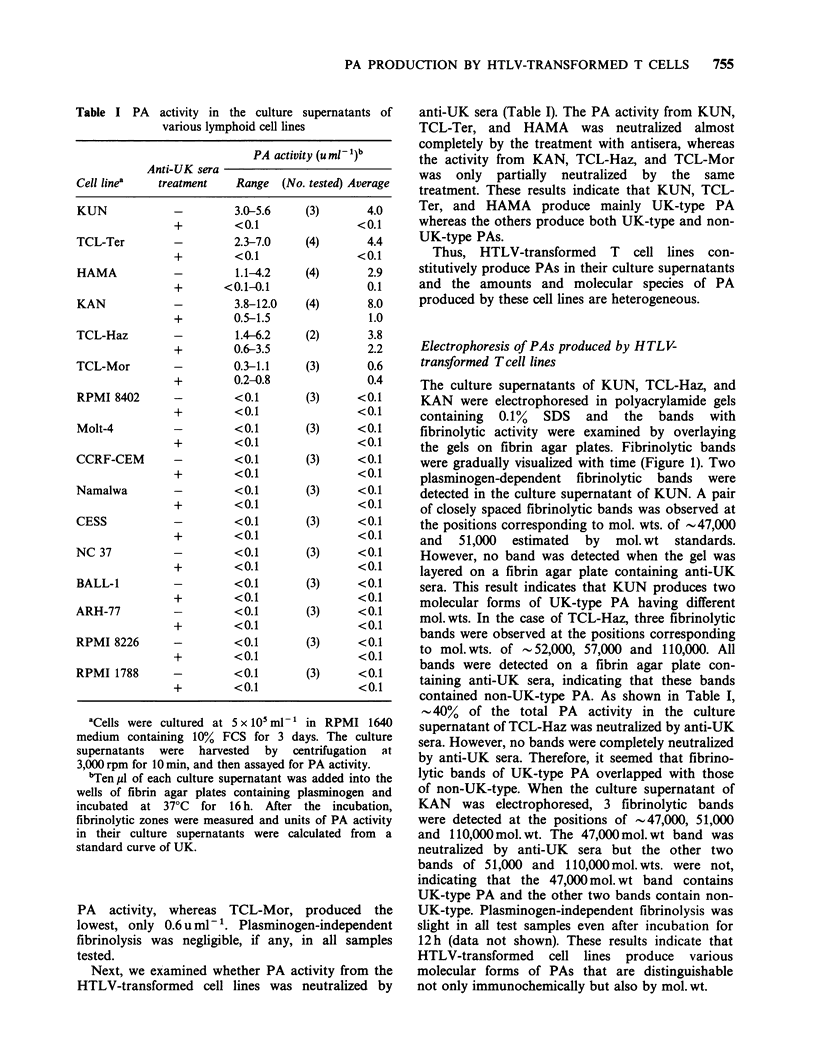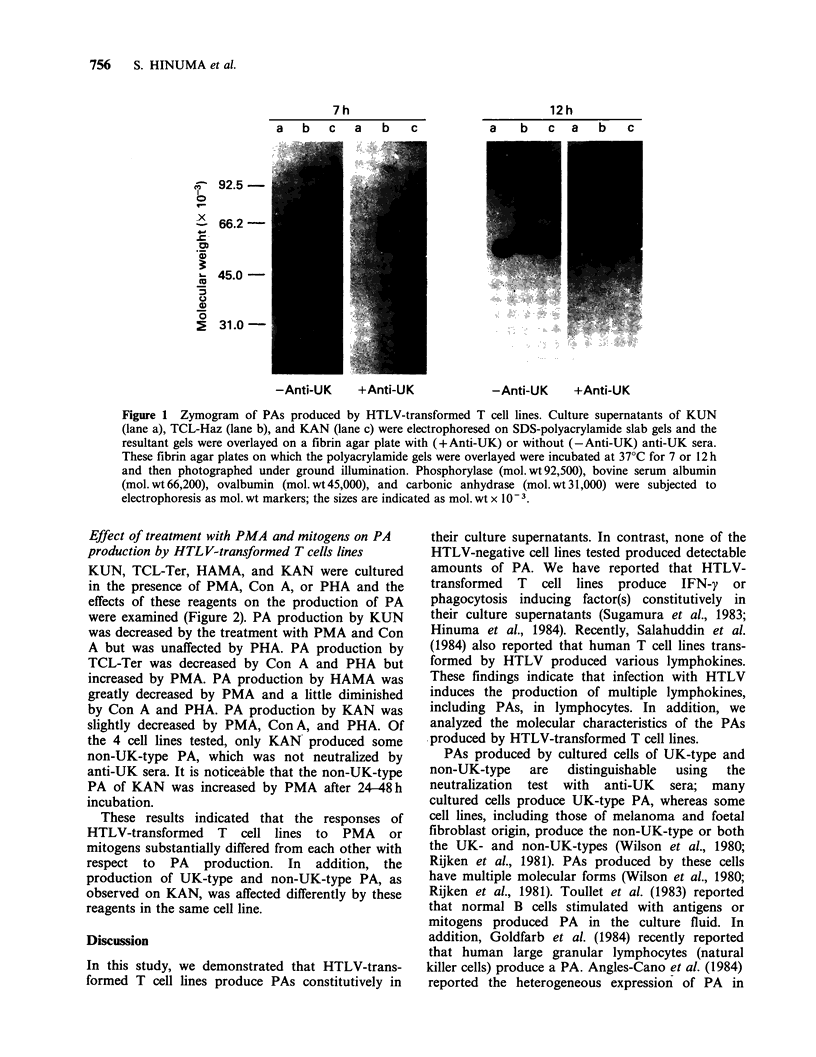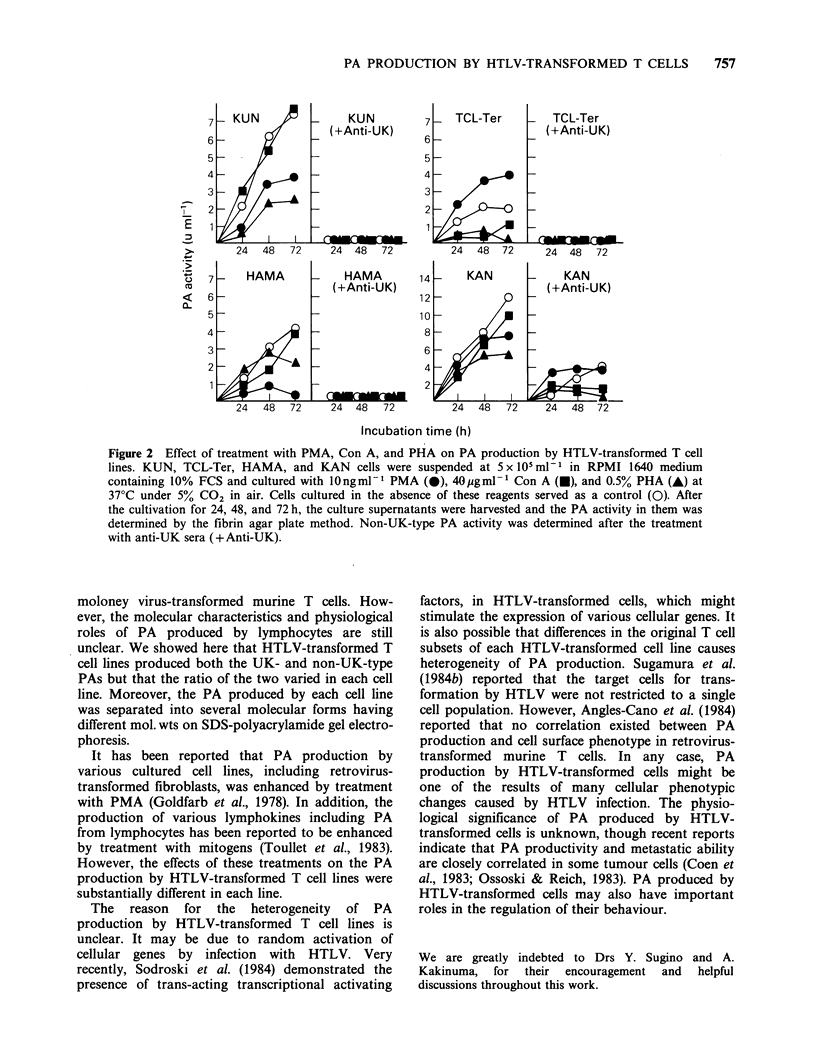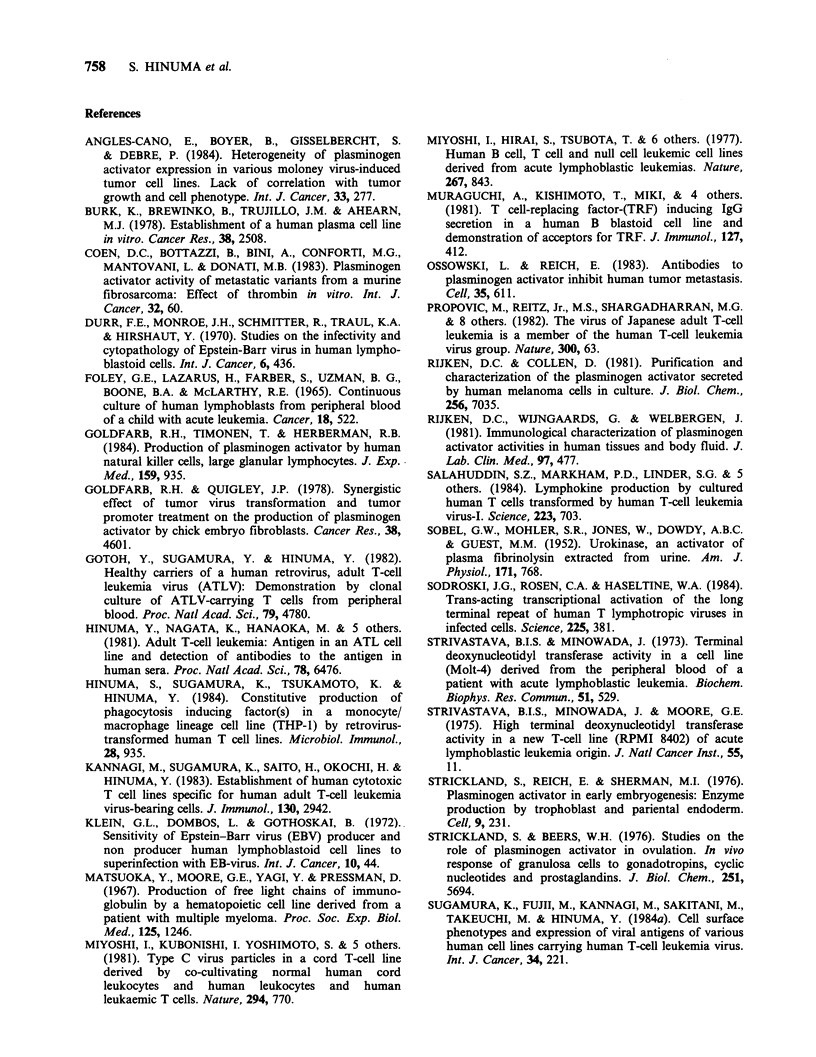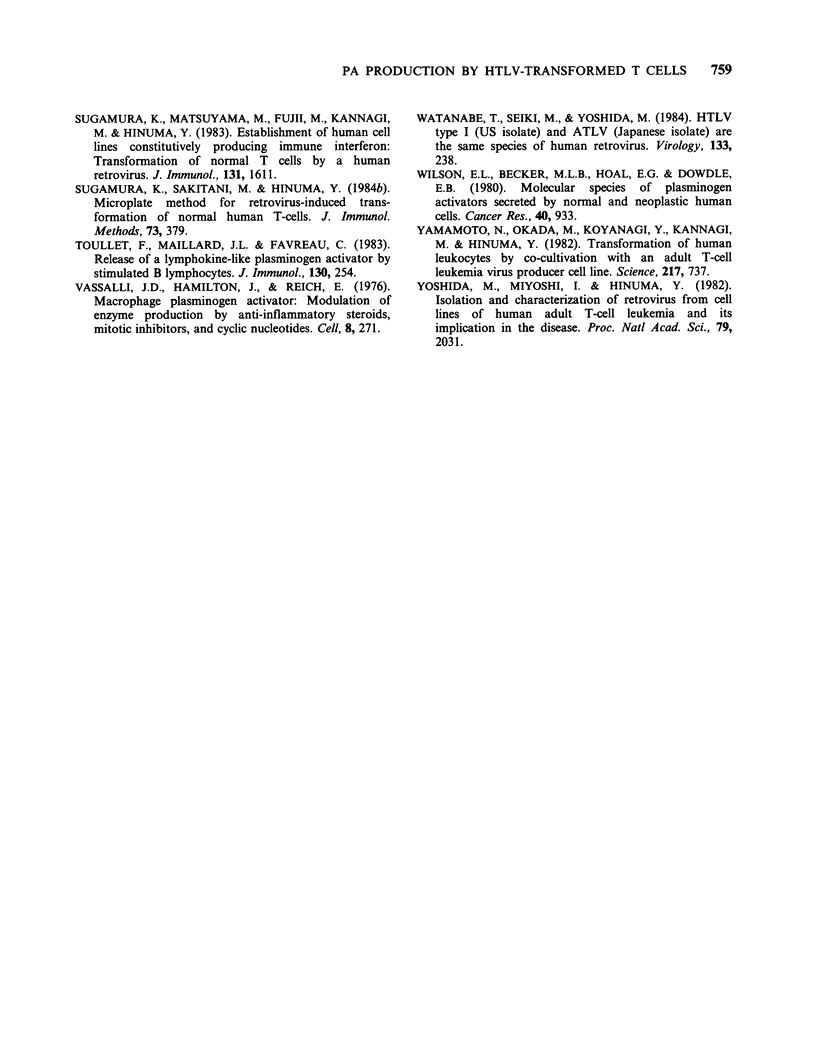# Production of plasminogen activators by human T-cell leukaemia virus-transformed human T cell lines.

**DOI:** 10.1038/bjc.1985.118

**Published:** 1985-06

**Authors:** S. Hinuma, S. Honda, K. Tsukamoto, K. Sugamura, Y. Hinuma

## Abstract

**Images:**


Br. J. Cancer (1985), **51**, 753–759

### Production of plasminogen activators by human T-cell leukaemia virus-transformed human T cell lines

S. Hinuma[1], S. Honda[1], K. Tsukamoto[1], K. Sugamura[2] & Y. Hinuma[2]

[1]*Biotechnology Laboratories, Central Research Division, Takeda Chemical Industries, Ltd., 17–85, Jusohonmachi 2-chome, Yodogawa-Ku, Osaka 532, and* [2]*the Institute for Virus Research, Kyoto University, Sakyo-Ku, Kyoto 606, Japan.*

**Summary** Six human T cell lines HAMA, KUN, KAN, TCL-Haz, TCL-Ter, and TCL-Mor, which were transformed by a retrovirus, human T-cell leukaemia virus (HTLV), constitutively produced plasminogen activators (PAs) in culture supernatants. The amount of PAs produced varied among the cell lines. The PAs were distinguished by immunochemical analysis between two types: urokinase (UK)-type and non-UK-type. KUN, TCL-Ter, and HAMA mainly produced UK-type PA, whereas the other cell lines produced both types. Thus, HTLV-transformed T cell lines differ in the quality and quantity of the PAs they produce. The PAs in the culture supernatants of each cell line were separated into several mol. w forms on sodium dodecyl sulfate polyacrylamide gel electrophoresis. The results indicate that the same cell line produces PAs of different mol. wt. PA production by these cell lines was affected by treatment with phorbol miristate acetate, concanavalin A, and phytohaemagglutinin; the effects were substantially different in each cell line. The data described here indicate that HTLV-transformed T cell lines constitutively produce PAs which are very heterogeneous in both quality and quantity.

Plasminogen activators (PAs) convert plasminogen to an active form, plasmin, which in turn degrades fibrin and other protein substrates. PAs have been found in various normal tissues, body fluids (Rijkin *et al.*, 1981), secretions (Sobel *et al.*, 1952), and cultured cells *in vitro* (Wilson *et al.*, 1980; Rijken & Collen, 1981). PAs exist in several molecular forms that are distinguishable on the basis of their biochemical and immunochemical characteristics (Rijken & Collen, 1981; Wilson *et al.*, 1980; Rijken *et al.*, 1981). PAs have important roles in extracellular fibrinolysis to remove fibrin deposit and in the regulation of various behaviour of normal and malignant cells including migration, tissue remodeling, and tissue destruction (Strickland & Beers, 1976; Strickland *et al.*, 1976; Vassali *et al.*, 1976).

Recently, a unique human C-type retrovirus, adult T-cell leukaemia (ATL) virus (ATLV), was demonstrated in leukaemic cells from patients with ATL in Japan (Hinuma *et al.*, 1981; Miyoshi *et al.*, 1981; Yoshida *et al.*, 1982). At present, it is known that ATLV is identical to human T-cell leukaemia virus-I (HTLV-I) originally isolated in a cutaneous T cell lymphoma cell line in the USA (Propovic *et al.*, 1982; Watanabe *et al.*, 1984). The virus can be transmitted to normal adult peripheral and umbilical cord blood leucocytes accompanied by transformation by the method of coculture with HTLV-producing cells (Yamamoto *et al.*, 1982; Sugamura *et al.*, 1983; Kannagi *et al.*, 1983). HTLV-bearing cell lines can also be established from leucocytes of patients with ATL or HTLV-carriers using interleukin 2 (IL 2) (Gotoh *et al.*, 1982; Sugamura *et al.*, 1984*a*). Cells transformed by HTLV possess proviral DNA sequences integrated into the cellular DNA (Yoshida *et al.*, 1982) and they express HTLV-associated antigens (Yamamoto *et al.*, 1982; Sugamura *et al.*, 1983; Kannagi *et al.*, 1983) in most cases and often produce virus-particles (Miyoshi *et al.*, 1981). Many HTLV- transformed cell lines have been established by these methods and we reported that some lines could produce lymphokines including interferon-$\gamma$ (IFN-$\gamma$) or phagocytosis inducing factor(s) (Sugamura *et al.*, 1983; Hinuma *et al.*, 1984). In this study, we examined the production of PA by HTLV-transformed T cell lines and found that they produced PAs constitutively in the culture supernatants. Furthermore, we found that the PAs produced by these cell lines were heterogeneous.

### Materials and methods

### Cell lines

HAMA, KUN, KAN, TCL-Haz, TCL-Ter and TCL-Mor were the HTLV-transformed cell lines used. HAMA was established from peripheral blood leucocytes of a healthy HTLV-carrier; initially it grew in the presence of IL 2, subsequently without IL 2. The other HTLV-transformed cell lines were established from normal peripheral blood leucocytes after coculture with X-ray-irradiated MT-2 cells as described previously (Yamamoto *et al.*, 1982; Sugamura *et al.*, 1983; Kannagi *et al.*, 1983). All

Correspondence: S. Hinuma.
Received 22 January 1985.

© The Macmillan Press Ltd., 1985

these lines were positive for HTLV-associated antigens and T cell surface markers, but negative for Epstein–Barr virus-associated antigens and surface immunoglobulins (Yamamoto *et al.*, 1982; Sugamura *et al.*, 1984*a*). Other HTLV-negative cell lines used were as follows. T cell lines; RPMI 8402 (Strivastava *et al.*, 1975), Molt-4 (Strivastava & Minowada, 1973), and CCRF-CEM (Foley *et al.*, 1965): B cell lines; Namalwa (Klein *et al.*, 1972), CESS (Muraguchi *et al.*, 1981), NC 37 (Durr *et al.*, 1970), and BALL-1 (Miyoshi *et al.*, 1977); myeloma cell lines; ARH-77 (Burk *et al.*, 1978) and RPMI 8226 (Matsuoka *et al.*, 1967). They were maintained at 37°C in RPMI 1640 (M.A. Bioproducts, Maryland, USA) supplemented with $100 \mu g ml^{-1}$ kanamycin, $2 mg ml^{-1}$ $NaHCO_3$, 25 mM N-2-hydroxyethylpiperazine-N′-2-ethanesulfonic acid (RPMI 1640 medium), and 10% foetal calf serum (FCS).

*Preparation of culture supernatants*

Cells were washed with RPMI 1640 medium containing 10% FCS, resuspended at $5 \times 10^5 ml^{-1}$ in the same medium and cultured at 37°C for 24–72 h in a volume of 1 ml in Linbro 24-well microplates (Flow Laboratories, Inc., Milwaukee, USA) under a humidified atmosphere of 5% $CO_2$ in air. In some experiments, cells were cultured in the presence of $40 \mu g ml^{-1}$ concanavalin A (Con A) (P-L Biochemicals, Inc., Milwaukee, USA), 0.5% phytohaemagglutinin (PHA) (Difco Laboratories, Detroit, USA), or $10 ng ml^{-1}$ phorbol miristate acetate (PMA). After the incubation, the culture supernatants were obtained by centrifugation at 3,000 rpm for 10 min.

*Assay for PA*

PA activity in the culture supernatants was determined by plasminogen-dependent fibrinolysis using fibrin agar plates. Fibrin agar plates were prepared as follows: 1.25% special noble agar (Difco Laboratories, Detroit, USA) dissolved in PBS (pH 7.4) and prewarmed at 42°C, was quickly mixed with bovine fibrinogen (Seikagaku Kogyo, Co., Ltd., Tokyo, Japan) at a final concentration of $2.0 mg ml^{-1}$, bovine plasminogen ($0.3 u ml^{-1}$) (Seikagaku Kogyo, Co., Ltd., Tokyo, Japan), and bovine thrombin ($0.06 u ml^{-1}$) (Mochida Pharmaceutical Co., Ltd., Tokyo, Japan). The mixture (9.6 ml) was poured onto a 9 cm plastic petri dish and incubated at 4°C for 2 h. After fibrin formation, wells (4 mm diameter) were punched in the fibrin agar plate. Ten μl of a test sample were added to the wells in duplicate. The plates were incubated at 37°C for 16 h and diameters of lytic zones were measured by calipers. Plasminogen-independent fibrinolysis was tested on plasminogen-free fibrin agar plates. PA activity was expressed in international units by reference to a urokinase (UK) (Green Cross, Co., Ltd., Osaka, Japan) standard curve.

*Treatment with anti-UK sera*

Five μl of rabbit anti-UK sera (Green Cross, Co., Ltd., Osaka, Japan) diluted 100-fold with PBS and an equal volume of a test sample were added to the wells of fibrin agar plates and incubated at 4°C for 1 h; normal rabbit sera served as a control. After being incubated at 4°C, the plates were incubated at 37°C for a further 16 h and the lytic area was measured. Less than 20 units of UK were completely neutralized by this treatment.

*Polyacrylamide gel electrophoresis of PAs*

Sodium dodecyl sulfate (SDS)-polyacrylamide slab gels were prepared with upper stacking gels of 4% acrylamide and lower resolving gels of 10% acrylamide containing 0.1% SDS. Twenty μl of a test sample treated with 0.1% SDS were applied to the gels and electrophoresed at 4°C for 16 h at 4 mA/plate. Phosphorylase A (mol. wt 92,500), bovine serum albumin (mol. wt 66,200), ovalubumin (mol. wt 45,000), and carbonic anhydrase (mol. wt 31,000) were electrophoresed as standard mol. wt markers separately from the test samples. After the electrophoresis, the gels were washed 4 times in 2.5% Triton X-100 and subsequently rinsed with distilled water. The gels were layered on a fibrin agar plate consisting of 1.25% agar, fibrinogen (2 mg), thrombin ($0.06 u ml^{-1}$), and plasminogen ($0.3 u ml^{-1}$) with or without rabbit anti-UK sera (100-fold dilution). These gels were then incubated at 37°C. Bands having PA activity were seen under dark-ground illumination as clear lytic zones in the opaque fibrin agar plate.

### Results

*PA activity in the culture supernatants of HTLV-transformed T cell lines*

PA activity in the culture supernatants of the 6 HTLV-transformed T cell lines and the ten HTLV-negative cell lines was tested (Table I). PA activity was detected in the culture supernatants of all the HTLV-transformed T cell lines. In contrast, no activity was detected in those of the HTLV-negative cell lines. Six HTLV-transformed T cell lines produced considerably different amounts of PA. KAN, produced the greatest amount, $\sim 8 u ml^{-1}$ of

**Table I** PA activity in the culture supernatants of various lymphoid cell lines

| Cell line[a] | Anti-UK sera treatment | PA activity (u ml$^{-1}$)[b] Range | (No. tested) | Average |
|---|---|---|---|---|
| KUN | – | 3.0–5.6 | (3) | 4.0 |
| | + | <0.1 | | <0.1 |
| TCL-Ter | – | 2.3–7.0 | (4) | 4.4 |
| | + | <0.1 | | <0.1 |
| HAMA | – | 1.1–4.2 | (4) | 2.9 |
| | + | <0.1–0.1 | | 0.1 |
| KAN | – | 3.8–12.0 | (4) | 8.0 |
| | + | 0.5–1.5 | | 1.0 |
| TCL-Haz | – | 1.4–6.2 | (2) | 3.8 |
| | + | 0.6–3.5 | | 2.2 |
| TCL-Mor | – | 0.3–1.1 | (3) | 0.6 |
| | + | 0.2–0.8 | | 0.4 |
| RPMI 8402 | – | <0.1 | (3) | <0.1 |
| | + | <0.1 | | <0.1 |
| Molt-4 | – | <0.1 | (3) | <0.1 |
| | + | <0.1 | | <0.1 |
| CCRF-CEM | – | <0.1 | (3) | <0.1 |
| | + | <0.1 | | <0.1 |
| Namalwa | – | <0.1 | (3) | <0.1 |
| | + | <0.1 | | <0.1 |
| CESS | – | <0.1 | (3) | <0.1 |
| | + | <0.1 | | <0.1 |
| NC 37 | – | <0.1 | (3) | <0.1 |
| | + | <0.1 | | <0.1 |
| BALL-1 | – | <0.1 | (3) | <0.1 |
| | + | <0.1 | | <0.1 |
| ARH-77 | – | <0.1 | (3) | <0.1 |
| | + | <0.1 | | <0.1 |
| RPMI 8226 | – | <0.1 | (3) | <0.1 |
| | + | <0.1 | | <0.1 |
| RPMI 1788 | – | <0.1 | (3) | <0.1 |
| | + | <0.1 | | <0.1 |

[a]Cells were cultured at $5 \times 10^5$ ml$^{-1}$ in RPMI 1640 medium containing 10% FCS for 3 days. The culture supernatants were harvested by centrifugation at 3,000 rpm for 10 min, and then assayed for PA activity.

[b]Ten $\mu$l of each culture supernatant was added into the wells of fibrin agar plates containing plasminogen and incubated at 37°C for 16 h. After the incubation, fibrinolytic zones were measured and units of PA activity in their culture supernatants were calculated from a standard curve of UK.

PA activity, whereas TCL-Mor, produced the lowest, only 0.6 u ml$^{-1}$. Plasminogen-independent fibrinolysis was negligible, if any, in all samples tested.

Next, we examined whether PA activity from the HTLV-transformed cell lines was neutralized by anti-UK sera (Table I). The PA activity from KUN, TCL-Ter, and HAMA was neutralized almost completely by the treatment with antisera, whereas the activity from KAN, TCL-Haz, and TCL-Mor was only partially neutralized by the same treatment. These results indicate that KUN, TCL-Ter, and HAMA produce mainly UK-type PA whereas the others produce both UK-type and non-UK-type PAs.

Thus, HTLV-transformed T cell lines constitutively produce PAs in their culture supernatants and the amounts and molecular species of PA produced by these cell lines are heterogeneous.

### *Electrophoresis of PAs produced by HTLV-transformed T cell lines*

The culture supernatants of KUN, TCL-Haz, and KAN were electrophoresed in polyacrylamide gels containing 0.1% SDS and the bands with fibrinolytic activity were examined by overlaying the gels on fibrin agar plates. Fibrinolytic bands were gradually visualized with time (Figure 1). Two plasminogen-dependent fibrinolytic bands were detected in the culture supernatant of KUN. A pair of closely spaced fibrinolytic bands was observed at the positions corresponding to mol. wts. of ~47,000 and 51,000 estimated by mol. wt standards. However, no band was detected when the gel was layered on a fibrin agar plate containing anti-UK sera. This result indicates that KUN produces two molecular forms of UK-type PA having different mol. wts. In the case of TCL-Haz, three fibrinolytic bands were observed at the positions corresponding to mol. wts. of ~52,000, 57,000 and 110,000. All bands were detected on a fibrin agar plate containing anti-UK sera, indicating that these bands contained non-UK-type PA. As shown in Table I, ~40% of the total PA activity in the culture supernatant of TCL-Haz was neutralized by anti-UK sera. However, no bands were completely neutralized by anti-UK sera. Therefore, it seemed that fibrinolytic bands of UK-type PA overlapped with those of non-UK-type. When the culture supernatant of KAN was electrophoresed, 3 fibrinolytic bands were detected at the positions of ~47,000, 51,000 and 110,000 mol. wt. The 47,000 mol. wt band was neutralized by anti-UK sera but the other two bands of 51,000 and 110,000 mol. wts. were not, indicating that the 47,000 mol. wt band contains UK-type PA and the other two bands contain non-UK-type. Plasminogen-independent fibrinolysis was slight in all test samples even after incubation for 12 h (data not shown). These results indicate that HTLV-transformed cell lines produce various molecular forms of PAs that are distinguishable not only immunochemically but also by mol. wt.

**Figure 1** Zymogram of PAs produced by HTLV-transformed T cell lines. Culture supernatants of KUN (lane a), TCL-Haz (lane b), and KAN (lane c) were electrophoresed on SDS-polyacrylamide slab gels and the resultant gels were overlayed on a fibrin agar plate with (+Anti-UK) or without (−Anti-UK) anti-UK sera. These fibrin agar plates on which the polyacrylamide gels were overlayed were incubated at 37°C for 7 or 12 h and then photographed under ground illumination. Phosphorylase (mol. wt 92,500), bovine serum albumin (mol. wt 66,200), ovalbumin (mol. wt 45,000), and carbonic anhydrase (mol. wt 31,000) were subjected to electrophoresis as mol. wt markers; the sizes are indicated as mol. wt $\times 10^{-3}$.

*Effect of treatment with PMA and mitogens on PA production by HTLV-transformed T cells lines*

KUN, TCL-Ter, HAMA, and KAN were cultured in the presence of PMA, Con A, or PHA and the effects of these reagents on the production of PA were examined (Figure 2). PA production by KUN was decreased by the treatment with PMA and Con A but was unaffected by PHA. PA production by TCL-Ter was decreased by Con A and PHA but increased by PMA. PA production by HAMA was greatly decreased by PMA and a little diminished by Con A and PHA. PA production by KAN was slightly decreased by PMA, Con A, and PHA. Of the 4 cell lines tested, only KAN produced some non-UK-type PA, which was not neutralized by anti-UK sera. It is noticeable that the non-UK-type PA of KAN was increased by PMA after 24–48 h incubation.

These results indicated that the responses of HTLV-transformed T cell lines to PMA or mitogens substantially differed from each other with respect to PA production. In addition, the production of UK-type and non-UK-type PA, as observed on KAN, was affected differently by these reagents in the same cell line.

### Discussion

In this study, we demonstrated that HTLV-transformed T cell lines produce PAs constitutively in their culture supernatants. In contrast, none of the HTLV-negative cell lines tested produced detectable amounts of PA. We have reported that HTLV-transformed T cell lines produce IFN-γ or phagocytosis inducing factor(s) constitutively in their culture supernatants (Sugamura *et al.*, 1983; Hinuma *et al.*, 1984). Recently, Salahuddin *et al.* (1984) also reported that human T cell lines transformed by HTLV produced various lymphokines. These findings indicate that infection with HTLV induces the production of multiple lymphokines, including PAs, in lymphocytes. In addition, we analyzed the molecular characteristics of the PAs produced by HTLV-transformed T cell lines.

PAs produced by cultured cells of UK-type and non-UK-type are distinguishable using the neutralization test with anti-UK sera; many cultured cells produce UK-type PA, whereas some cell lines, including those of melanoma and foetal fibroblast origin, produce the non-UK-type or both the UK- and non-UK-types (Wilson *et al.*, 1980; Rijken *et al.*, 1981). PAs produced by these cells have multiple molecular forms (Wilson *et al.*, 1980; Rijken *et al.*, 1981). Toullet *et al.* (1983) reported that normal B cells stimulated with antigens or mitogens produced PA in the culture fluid. In addition, Goldfarb *et al.* (1984) recently reported that human large granular lymphocytes (natural killer cells) produce a PA. Angles-Cano *et al.* (1984) reported the heterogeneous expression of PA in

**Figure 2** Effect of treatment with PMA, Con A, and PHA on PA production by HTLV-transformed T cell lines. KUN, TCL-Ter, HAMA, and KAN cells were suspended at $5\times10^5$ ml$^{-1}$ in RPMI 1640 medium containing 10% FCS and cultured with 10 ng ml$^{-1}$ PMA (●), 40 $\mu$g ml$^{-1}$ Con A (■), and 0.5% PHA (▲) at 37°C under 5% $CO_2$ in air. Cells cultured in the absence of these reagents served as a control (○). After the cultivation for 24, 48, and 72 h, the culture supernatants were harvested and the PA activity in them was determined by the fibrin agar plate method. Non-UK-type PA activity was determined after the treatment with anti-UK sera (+Anti-UK).

moloney virus-transformed murine T cells. However, the molecular characteristics and physiological roles of PA produced by lymphocytes are still unclear. We showed here that HTLV-transformed T cell lines produced both the UK- and non-UK-type PAs but that the ratio of the two varied in each cell line. Moreover, the PA produced by each cell line was separated into several molecular forms having different mol. wts on SDS-polyacrylamide gel electrophoresis.

It has been reported that PA production by various cultured cell lines, including retrovirus-transformed fibroblasts, was enhanced by treatment with PMA (Goldfarb *et al.*, 1978). In addition, the production of various lymphokines including PA from lymphocytes has been reported to be enhanced by treatment with mitogens (Toullet *et al.*, 1983). However, the effects of these treatments on the PA production by HTLV-transformed T cell lines were substantially different in each line.

The reason for the heterogeneity of PA production by HTLV-transformed T cell lines is unclear. It may be due to random activation of cellular genes by infection with HTLV. Very recently, Sodroski *et al.* (1984) demonstrated the presence of trans-acting transcriptional activating factors, in HTLV-transformed cells, which might stimulate the expression of various cellular genes. It is also possible that differences in the original T cell subsets of each HTLV-transformed cell line causes heterogeneity of PA production. Sugamura *et al.* (1984*b*) reported that the target cells for transformation by HTLV were not restricted to a single cell population. However, Angles-Cano *et al.* (1984) reported that no correlation existed between PA production and cell surface phenotype in retrovirus-transformed murine T cells. In any case, PA production by HTLV-transformed cells might be one of the results of many cellular phenotypic changes caused by HTLV infection. The physiological significance of PA produced by HTLV-transformed cells is unknown, though recent reports indicate that PA productivity and metastatic ability are closely correlated in some tumour cells (Coen *et al.*, 1983; Ossoski & Reich, 1983). PA produced by HTLV-transformed cells may also have important roles in the regulation of their behaviour.

We are greatly indebted to Drs Y. Sugino and A. Kakinuma, for their encouragement and helpful discussions throughout this work.

### References

ANGLES-CANO, E., BOYER, B., GISSELBERCHT, S. & DEBRE, P. (1984). Heterogeneity of plasminogen activator expression in various moloney virus-induced tumor cell lines. Lack of correlation with tumor growth and cell phenotype. *Int. J. Cancer*, **33**, 277.

BURK, K., BREWINKO, B., TRUJILLO, J.M. & AHEARN, M.J. (1978). Establishment of a human plasma cell line *in vitro*. *Cancer Res.*, **38**, 2508.

COEN, D.C., BOTTAZZI, B., BINI, A., CONFORTI, M.G., MANTOVANI, L. & DONATI, M.B. (1983). Plasminogen activator activity of metastatic variants from a murine fibrosarcoma: Effect of thrombin *in vitro*. *Int. J. Cancer*, **32**, 60.

DURR, F.E., MONROE, J.H., SCHMITTER, R., TRAUL, K.A. & HIRSHAUT, Y. (1970). Studies on the infectivity and cytopathology of Epstein-Barr virus in human lymphoblastoid cells. *Int. J. Cancer*, **6**, 436.

FOLEY, G.E., LAZARUS, H., FARBER, S., UZMAN, B. G., BOONE, B.A. & McLARTHY, R.E. (1965). Continuous culture of human lymphoblasts from peripheral blood of a child with acute leukemia. *Cancer*, **18**, 522.

GOLDFARB, R.H., TIMONEN, T. & HERBERMAN, R.B. (1984). Production of plasminogen activator by human natural killer cells, large glanular lymphocytes. *J. Exp. Med.*, **159**, 935.

GOLDFARB, R.H. & QUIGLEY, J.P. (1978). Synergistic effect of tumor virus transformation and tumor promoter treatment on the production of plasminogen activator by chick embryo fibroblasts. *Cancer Res.*, **38**, 4601.

GOTOH, Y., SUGAMURA, Y. & HINUMA, Y. (1982). Healthy carriers of a human retrovirus, adult T-cell leukemia virus (ATLV): Demonstration by clonal culture of ATLV-carrying T cells from peripheral blood. *Proc. Natl Acad. Sci.*, **79**, 4780.

HINUMA, Y., NAGATA, K., HANAOKA, M. & 5 others. (1981). Adult T-cell leukemia: Antigen in an ATL cell line and detection of antibodies to the antigen in human sera. *Proc. Natl Acad. Sci.*, **78**, 6476.

HINUMA, S., SUGAMURA, K., TSUKAMOTO, K. & HINUMA, Y. (1984). Constitutive production of phagocytosis inducing factor(s) in a monocyte/macrophage lineage cell line (THP-1) by retrovirus-transformed human T cell lines. *Microbiol. Immunol.*, **28**, 935.

KANNAGI, M., SUGAMURA, K., SAITO, H., OKOCHI, H. & HINUMA, Y. (1983). Establishment of human cytotoxic T cell lines specific for human adult T-cell leukemia virus-bearing cells. *J. Immunol.*, **130**, 2942.

KLEIN, G.L., DOMBOS, L. & GOTHOSKAI, B. (1972). Sensitivity of Epstein–Barr virus (EBV) producer and non producer human lymphoblastoid cell lines to superinfection with EB-virus. *Int. J. Cancer*, **10**, 44.

MATSUOKA, Y., MOORE, G.E., YAGI, Y. & PRESSMAN, D. (1967). Production of free light chains of immunoglobulin by a hematopoietic cell line derived from a patient with multiple myeloma. *Proc. Soc. Exp. Biol. Med.*, **125**, 1246.

MIYOSHI, I., KUBONISHI, I. YOSHIMOTO, S. & 5 others. (1981). Type C virus particles in a cord T-cell line derived by co-cultivating normal human cord leukocytes and human leukocytes and human leukaemic T cells. *Nature*, **294**, 770.

MIYOSHI, I., HIRAI, S., TSUBOTA, T. & 6 others. (1977). Human B cell, T cell and null cell leukemic cell lines derived from acute lymphoblastic leukemias. *Nature*, **267**, 843.

MURAGUCHI, A., KISHIMOTO, T., MIKI, & 4 others. (1981). T cell-replacing factor-(TRF) inducing IgG secretion in a human B blastoid cell line and demonstration of acceptors for TRF. *J. Immunol.*, **127**, 412.

OSSOWSKI, L. & REICH, E. (1983). Antibodies to plasminogen activator inhibit human tumor metastasis. *Cell*, **35**, 611.

PROPOVIC, M., REITZ, Jr., M.S., SHARGADHARRAN, M.G. & 8 others. (1982). The virus of Japanese adult T-cell leukemia is a member of the human T-cell leukemia virus group. *Nature*, **300**, 63.

RIJKEN, D.C. & COLLEN, D. (1981). Purification and characterization of the plasminogen activator secreted by human melanoma cells in culture. *J. Biol. Chem.*, **256**, 7035.

RIJKEN, D.C., WIJNGAARDS, G. & WELBERGEN, J. (1981). Immunological characterization of plasminogen activator activities in human tissues and body fluid. *J. Lab. Clin. Med.*, **97**, 477.

SALAHUDDIN, S.Z., MARKHAM, P.D., LINDER, S.G. & 5 others. (1984). Lymphokine production by cultured human T cells transformed by human T-cell leukemia virus-I. *Science*, **223**, 703.

SOBEL, G.W., MOHLER, S.R., JONES, W., DOWDY, A.B.C. & GUEST, M.M. (1952). Urokinase, an activator of plasma fibrinolysin extracted from urine. *Am. J. Physiol.*, **171**, 768.

SODROSKI, J.G., ROSEN, C.A. & HASELTINE, W.A. (1984). Trans-acting transcriptional activation of the long terminal repeat of human T lymphotropic viruses in infected cells. *Science*, **225**, 381.

STRIVASTAVA, B.I.S. & MINOWADA, J. (1973). Terminal deoxynucleotidyl transferase activity in a cell line (Molt-4) derived from the peripheral blood of a patient with acute lymphoblastic leukemia. *Biochem. Biophys. Res. Commun.*, **51**, 529.

STRIVASTAVA, B.I.S., MINOWADA, J. & MOORE, G.E. (1975). High terminal deoxynucleotidyl transferase activity in a new T-cell line (RPMI 8402) of acute lymphoblastic leukemia origin. *J. Natl Cancer Inst.*, **55**, 11.

STRICKLAND, S., REICH, E. & SHERMAN, M.I. (1976). Plasminogen activator in early embryogenesis: Enzyme production by trophoblast and pariental endoderm. *Cell*, **9**, 231.

STRICKLAND, S. & BEERS, W.H. (1976). Studies on the role of plasminogen activator in ovulation. *In vivo* response of granulosa cells to gonadotropins, cyclic nucleotides and prostaglandins. *J. Biol. Chem.*, **251**, 5694.

SUGAMURA, K., FUJII, M., KANNAGI, M., SAKITANI, M., TAKEUCHI, M. & HINUMA, Y. (1984*a*). Cell surface phenotypes and expression of viral antigens of various human cell lines carrying human T-cell leukemia virus. *Int. J. Cancer*, **34**, 221.

SUGAMURA, K., MATSUYAMA, M., FUJII, M., KANNAGI, M. & HINUMA, Y. (1983). Establishment of human cell lines constitutively producing immune interferon: Transformation of normal T cells by a human retrovirus. *J. Immunol.*, **131**, 1611.

SUGAMURA, K., SAKITANI, M. & HINUMA, Y. (1984*b*). Microplate method for retrovirus-induced transformation of normal human T-cells. *J. Immunol. Methods*, **73**, 379.

TOULLET, F., MAILLARD, J.L. & FAVREAU, C. (1983). Release of a lymphokine-like plasminogen activator by stimulated B lymphocytes. *J. Immunol.*, **130**, 254.

VASSALLI, J.D., HAMILTON, J., & REICH, E. (1976). Macrophage plasminogen activator: Modulation of enzyme production by anti-inflammatory steroids, mitotic inhibitors, and cyclic nucleotides. *Cell*, **8**, 271.

WATANABE, T., SEIKI, M., & YOSHIDA, M. (1984). HTLV type I (US isolate) and ATLV (Japanese isolate) are the same species of human retrovirus. *Virology*, **133**, 238.

WILSON, E.L., BECKER, M.L.B., HOAL, E.G. & DOWDLE, E.B. (1980). Molecular species of plasminogen activators secreted by normal and neoplastic human cells. *Cancer Res.*, **40**, 933.

YAMAMOTO, N., OKADA, M., KOYANAGI, Y., KANNAGI, M. & HINUMA, Y. (1982). Transformation of human leukocytes by co-cultivation with an adult T-cell leukemia virus producer cell line. *Science*, **217**, 737.

YOSHIDA, M., MIYOSHI, I. & HINUMA, Y. (1982). Isolation and characterization of retrovirus from cell lines of human adult T-cell leukemia and its implication in the disease. *Proc. Natl Acad. Sci.*, **79**, 2031.